# Mortality outcomes in 35,433 patients admitted for acute haemorrhagic stroke in Australia: A population-linkage study

**DOI:** 10.1016/j.ijcrp.2024.200258

**Published:** 2024-03-20

**Authors:** Arielle Chin-yu Hsu, Vijayatubini Vijayarajan, Yeu-Yao Cheng, Matthew Wei Shun Shu, Karice Hyun, Vincent Chow, David Brieger, Leonard Kritharides, Austin Chin Chwan Ng

**Affiliations:** Department of Cardiology, Concord Hospital, The University of Sydney, 1 Hospital Road, Concord, 2139, NSW, Australia

## Abstract

**Background:**

Haemorrhagic stroke (HS) is an important cardiovascular cause of mortality worldwide. Trends in admission rates and outcomes, and predictors of outcomes, post-HS in Australia remain unclear.

**Methods:**

All New South Wales residents, Australia, hospitalized with HS from 2002 to 2017 were identified from the Admitted-Patient-Data-Collection database. Admission rates were adjusted to population size by sex, age-groups and calendar-year. Mortality was tracked from the death registry to 31-Dec-2018 and adjusted for admission calendar-year, age, gender, referral source, surgical evacuation following HS and comorbidities.

**Results:**

The cohort comprised 35,433 patients (51.1% males). Overall age-adjusted mean(±SD) admission rates were higher for males (63.6 ± 6.2 vs 49.9 ± 4.4 admissions-per-100,000-persons-per-annum). Annual admission rates declined for both sexes from 2002 to 2017 especially in those ≥60yo. In-hospital and 1-year mortality rates were higher for females than males (25.0% vs 20.0% and 40.6% vs 35.9% respectively, all p < 0.001). Adjusted in-hospital and 1-year mortality declined for men and women, overall decreasing by 45% (odds ratio 0.55, 95% confidence interval [CI] = 0.47–0.64), and 31% (hazard ratio 0.69,95%CI = 0.63–0.76) respectively between 2002 and 2017. Independent predictors of increased in-hospital and 1-year mortality included increasing age and Charlson comorbidity index, while male sex, a history of hyperlipidaemia and current smoking, and surgical evacuation following HS were associated with reduced mortality (all p < 0.001).

**Conclusion:**

HS incidence increases markedly with age. Although age-adjusted HS admission rates and post HS mortality have fallen, HS remains associated with high early and 1-year mortality, with females consistently associated with worse outcomes. Strategies to improve outcomes of these patients remain a clinical priority.

## 1 Introduction

Stroke continues to be a leading cause of disability and poses as one of the greatest disease-related economic burdens in Australia [1]. Haemorrhagic strokes (HS), including intracranial haemorrhages (ICH) and subarachnoid haemorrhages (SAH), account for 37.6% of strokes globally, with incidence rates of 41.8 and 14.5 per 100,000 persons, respectively [2]. A previous Australian study analysed statewide admissions from 2005 to 2013 in New South Wales (NSW) and reported hospital admission rates of 16 and 9 per 100,000 persons for ICH and SAH, respectively [3]. More recent HS hospitalisation trends in Australia remain unclear.

HS has the highest mortality risk compared with other stroke types, with overseas crude mortality rates post-HS ranging from 32-38%–68% [[4], [5], [6]]. Global studies report a downward trend in crude mortality post-HS [7]. In Australia, crude mortality post-HS has also been declining [3,8,9]. Despite comorbidities in patients with HS commonly reported in the literature, few population-level studies have adjusted for comorbidities when analysing mortality outcomes post-HS [[10], [11], [12]]. A detailed understanding of the underlying long-term temporal trends in hospitalisation rates and outcomes, coupled with identifying specific groups at risk of adverse outcomes, will allow targeted escalation of primary prevention and stroke management.

The present study was performed with the following aims: 1) to determine trends in the prevalence of patients admitted with acute HS in NSW, the most populous state in Australia; and, 2) to determine the mortality trends following acute HS over a 16-year study period; and, 3) to identify predictors of adverse outcome.

## 2 Methods

### 2.1 Study population

The Centre-for-Health-Record-Linkage (CHeReL) is one of the largest data linkage systems in Australia containing linked health data for residents in the state of NSW [13]. The Admission-Patient-Data-Collection (APDC) database, which forms part of the CHeReL-derived master linkage key databases, includes ≥97% of all healthcare facilities in the state. We extracted data from the APDC database on consecutive admissions with a primary diagnosis of acute HS between 1-January-2002 and 31-December-2018 coded as I60, I61 or I62 under the International Statistical Classification of Diseases, 10th Revision Australian-Modification (ICD-10AM) coding system (Supplementary Table 1). For this study, cases were limited to only NSW state residents to minimize incomplete tracking (Supplementary Fig. 1 illustrates study cohort derivation).

### 2.2 Data sources

Data variables obtained from the APDC database include time/date of admission, age, sex, country of birth, referral source for admission, length of hospital stay, whether patient was admitted to intensive care unit (ICU), and whether surgical evacuation for ICH was performed during admission (see Supplementary Table 1 for the relevant Australian Classification of Health Interventions procedural codes).

The primary and all secondary diagnoses (potentially up to 50 secondary diagnoses) associated with each admission were retrieved from the APDC database. Comorbidities of interest extracted for this study (Supplementary Table 1 for ICD-10AM codes) are described in Table 1. The overall comorbid status of each patient was semi-quantified using the Charlson comorbidity index (CCI) which is a summation score based on 17 medical conditions with varying assigned weights (non-age-adjusted) [14]. A value of 0 indicates no comorbidity, while higher values represent an increasing burden of comorbid illnesses [14].

**Table 1:** Characteristics of cohort stratified by sex.

	Total cohort	Female	Male	
Parameters	N = 35,433	N = 17,323 (48.9)	N = 18,110 (51.1)	p-value
Age (years)	69.1 ± 17.7	70.4 ± 17.8	67.9 ± 17.5	
Median (25-75th IQ range)	74 (58–83)	75 (59–84)	72 (58–81)	<0.001
Length of admission (days)	8.6 ± 13.2	8.7 ± 13.1	9.6 ± 13.2	
Median (25-75th IQ range)	4 (1–11)	4 (1–11)	4 (1–11)	0.47
ICU admission	6675 (18.8)	3217 (18.6)	3458 (19.1)	0.21
Surgical evacuation of ICH	3674 (10.4)	1258 (7.3)	2416 (13.3)	<0.001
**Referral source**				
Emergency department	29,995 (84.7)	14,714 (84.9)	15,281 (84.4)	0.02
Physician-referred	1882 (5.3)	851 (4.9)	1031 (5.7)	
Hospital/community facility	3265 (9.2)	1615 (9.3)	1650 (9.1)	
Other	229 (0.6)	110 (0.6)	119 (0.7)	
Not known	62 (0.2)	33 (0.2)	29 (0.2)	
**Country of birth**				
Australia/New Zealand	23,349 (65.9)	11,841 (68.4)	11,508 (63.5)	<0.001
Mela-/Micro-/Poly-nesia	643 (1.8)	314 (1.8)	329 (1.8)	
Antarctica	4 (0.01)	1 (0.01)	3 (0.02)	
Europe	5620 (15.9)	2496 (14.4)	3124 (17.3)	
North Africa/Middle East	1039 (2.9)	407 (2.3)	632 (3.5)	
Asia	3242 (9.1)	1561 (9.0)	1681 (9.3)	
America	368 (1.0)	160 (0.9)	208 (1.1)	
Sub-Saharan Africa	249 (0.7)	124 (0.7)	125 (0.7)	
Not known	919 (2.6)	419 (2.4)	500 (2.8)	
**Comorbid conditions**				
Prior stroke^a^	3071 (8.7)	1726 (10.0)	1345 (7.4)	<0.001
Ischaemic/TIA	1257 (3.5)	732 (4.2)	525 (3.0)	<0.001
Haemorrhagic	1969 (5.6)	1096 (6.3)	873 (4.8)	<0.001
Ischaemic heart disease	1523 (4.3)	645 (3.7)	878 (4.8)	<0.001
Prior PCI/CABG	700 (2.0)	167 (1.0)	533 (2.9)	<0.001
Congestive cardiac failure	900 (2.5)	493 (2.8)	407 (2.2)	<0.001
Peripheral vascular disease	807 (2.3)	384 (2.2)	423 (2.3)	0.46
Valvular heart disease	259 (0.7)	127 (0.7)	132 (0.7)	1.00
Prosthetic heart valve	339 (1.0)	120 (0.7)	219 (1.2)	<0.001
Atrial fibrillation/flutter	3177 (9.0)	1524 (8.8)	1653 (9.1)	0.28
**Cardiac risk factors**				
Hypertension	16,586 (46.8)	8211 (47.4)	8375 (46.2)	0.03
Hyperlipidaemia	1605 (4.5)	732 (4.2)	873 (4.8)	0.007
Diabetes	4623 (13.0)	1882 (10.9)	2741 (15.1)	<0.001
Current smoker	3590 (10.1)	1549 (8.9)	2041 (11.3)	<0.001
Malignancy	1255 (3.5)	460 (2.7)	795 (4.4)	<0.001
Chronic pulmonary disease	818 (2.3)	392 (2.3)	426 (2.4)	0.60
Neurodegenerative disease^a^	2270 (6.4)	1212 (7.0)	1058 (5.8)	<0.001
Chronic kidney disease	750 (2.1)	256 (1.5)	494 (2.7)	<0.001
CCI score^b^	1.4 ± 1.9	1.2 ± 1.7	1.5 ± 2.0	
Median (25-75th IQ range)	1 (0–2)	1 (0–2)	1 (0–2)	<0.001

Plus-minus values represent mean ± standard deviation. All others represent numbers of patients with values in brackets representing percentages.

CABG, coronary artery bypass graft; CCI, Charlson comorbidity index; ICH, intracranial haemorrhage; ICU, intensive care unit; IQ, interquartile; PCI, percutaneous coronary intervention; TIA, transient ischaemic attack.

a Prior stroke includes ischaemic, haemorrhagic and/or TIA pre-2002 (a patient might have more than one type of prior strokes). Neurodegenerative disease includes dementia, central nervous systemic atrophies, Parkinson's disease, basal ganglia degeneration, and/or nervous systemic degenerative diseases.

b Conditions included in the Charlson comorbidity index include myocardial infarction, congestive cardiac failure, peripheral vascular disease, stroke, dementia, chronic pulmonary disease, connective tissue disease, peptic ulcer disease, liver disease (mild vs. moderate to severe), diabetes (with or without organ damage), hemiplegia, moderate to severe renal disease, any tumour (within past 5 years), lymphoma, leukemia, metastatic solid tumour, and acquired immunodeficiency syndrome.

### 2.3 Study outcome

The primary outcome was all-cause mortality tracked from the statewide death registry database held by CHeReL. The use of a statewide death registry to obtain our primary outcome is advantageous as non-captured deaths during this period were estimated to be only 0.6% based on known migration rates [15]. The final end-of-study follow-up date was set at 31-December-2018.

### 2.4 Statistical analysis

The study cohort was based on index acute HS admission between 1-January-2002 and 31-December-2017 (for patients who had recurrent HS presentations during the study period, only their index admission was included). The age-adjusted admission rates for a specific calendar-year were calculated by dividing the total index HS patients in each age category (0–9, 10–19, 20–29, 30–39, 40–49, 50–59, 60–69, 70–79, >80 years old [yo]) for that year by the year-specific total state population in corresponding age groups respectively. The NSW state population for each year was obtained from publicly available data provided by the Australian Bureau of Statistics [15]. All analyses were stratified by sex and presented as per-100,000-persons. Linear regression analysis was used to assess temporal trends.

All continuous variables were expressed as mean ± standard deviation, unless otherwise stated, and categorical data given in frequency and percentages. Comparison between groups used unpaired *t*-test for continuous variables and χ^2^ test or Fisher's exact test for dichotomous variables. To determine if mortality has improved over the course of the study period, mortality rates of each subsequent calendar-year were compared to year-2002 as reference. Cumulative mortality time-points of interest were pre-specified at in-hospital, 30-day, 3-month, 6-month, and 1-year. To assess mortality trends during study period, the time-points of interest were limited to in-hospital and 1-year. Binary logistic regression was used to compare differences in in-hospital mortality, while Cox proportional hazards regression was used to compare mortality at 1-year. Pre-specified univariables for consideration include calendar-year of admission, age, sex, referral source for admission, surgical evacuation of ICH, cardiovascular and noncardiovascular comorbidities, and CCI. Only univariables with P < 0.05 were included in the multivariable analysis. There were no missing values in any of the above analysis. A 2-tailed probability value of <0.05 was considered statistically significant. All analysis was performed using SPSS v23 (IBM, USA) and Prism 8 (GraphPad Software, CA, USA).

The NSW Population and Health Services Research Ethics Committee, reference number: 2013/09/479, granted ethics approval to conduct the study and a waiver of the usual requirement for the consent of the individual to the use of their health information. All patient data were de-identified and analysed anonymously.

## 3 Results

### 3.1 Temporal trends in admission cases

**Fig. 1:**
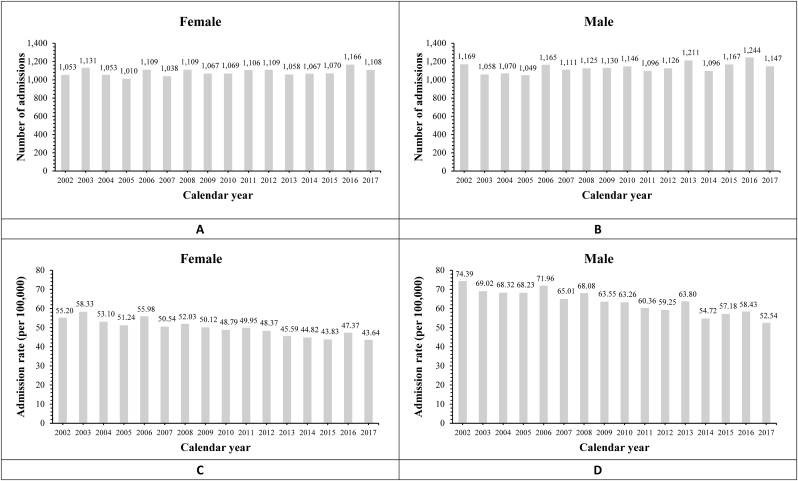
Volumes and age-adjusted admission rates of haemorrhagic stroke stratified by sex. Figure shows the annual volume and age-adjusted haemorrhagic stroke admissions between 1-January-2002 and 31-December-2017. The patient population was stratified by calendar year and sex. For each patient, only their index hospital admission for a stroke was included. Panel A and B shows the annual total admission caseload during the study period stratified by females and males respectively. Linear regression analysis was used to analyse temporal trends in admissions (female: r^2^ 0.06, p = 0.06; male: r^2^ 0.21, p = 0.04). Panel C and D shows the female and male respectively age-adjusted hospital admission rates during the study period. Admission rates are represented as per-100,000-person and adjusted for sex and age in decades (0–9, 10–19, 20–29, 30–39, 40–49, 50–59, 60–69, 70–79, >80 years) for each calendar year. The admission rates were calculated as the number of haemorrhagic stroke admissions divided by the sex and age group-specific New South Wales (NSW) population size for each calendar year. NSW population data was obtained from publicly available data from the Australian Bureau of Statistics (ABS). Linear regression analysis was used to analyse temporal trends in admission rates (female: r^2^ 0.82, p < 0.001; male: r^2^ 0.84, p < 0.001) (see Supplementary Table 2 for full analysis).

Between 2002 and 2017, a total of 35,433 patients were admitted with an index acute HS. The overall mean admission rate was 2215 ± 76 persons per-annum. Fig. 1 shows the annual admission volumes and age adjusted admission rates stratified by sex during the study period. After adjusting for calendar year, sex, and age-specific changes in NSW population size, the admission rates for males and females averaged 63.63 ± 6.22 and 49.93 ± 4.35 admissions-per-100,000-persons-per-annum respectively (Supplementary Table 2). Linear regression analysis showed the adjusted admission rates for both sexes declined significantly during the study period (both p < 0.001) (Fig. 1C and D, Supplementary Table 2).

When stratified by age categories, admission rates progressively increase with increasing age, with consistently highest admission rates in patients >80yo for both males and females (male: 298.9 ± 30.1 vs female: 240.6 ± 18.8 admissions-per-100,000-persons-per-annum) (Supplementary Tables 3 and 4). However, admission rates for older age groups declined the most significantly between 2002 and 2017. In females, admission rates for patients aged 60–69, 70–79 and > 80yo declined significantly (p = 0.02, p < 0.001, p < 0.001, respectively). Similarly, in males, admission rates for patients aged over 60yo also fell significantly during the study period (all p < 0.001). Contrastingly, though admission rates in younger patients of both sexes also fell in certain age categories, the decline was not as pronounced as observed in older age categories, with some younger age categories showing no change in admission rates between 2002 and 2017, likely a consequence of smaller admission volume.

### 3.2 Characteristics of study cohort

Table 1 details the study cohort's characteristics (51.1% male; mean age: 69.1 ± 17.7yo). Females were more likely to have had a prior stroke compared to males (10.0% vs 7.4%, p < 0.001). The rates of several other comorbidities and cardiovascular risk factors differed significantly between the sexes as shown in Table 1.

The mean admission duration was 8.6 ± 13.2 days and 18.8% of patients required ICU admission, with no significant differences between sexes. Males were more likely to undergo surgical evacuation for ICH during admission compared to females (13.3% vs 7.3%, p < 0.001). Further demographic information is available in Table 1.

## 4 Crude mortality outcomes

Supplementary Table 5 illustrates the baseline characteristics of patients who died in-hospital compared to those who were discharged alive. As expected, patients who died were older, proportionally more females than males and had greater comorbidity burden. The Kaplan-Meier survival curve following admission for haemorrhagic stroke is shown in Supplementary Fig. 2. The cumulative mortality rates stratified by sex and calendar year are shown in Supplementary Tables 6 and 7 Overall the cumulative in-hospital, 30-day, 3-month, 6-month and 1-year mortality rates were 20.0, 26.6, 30.4, 32.7 and 35.9% for males respectively, while the rates at all time-points were significantly higher in females at 25.0, 32.9, 35.9, 38.0 and 40.6% respectively (all p < 0.001). Supplementary Fig. 3 shows the Kaplan-Meier survival curves up to 1-year following admission for HS for calendar-years 2002, 2010 and 2017 highlighting the progressive improved survival during the study period. However, for both sexes, though crude mortality at all time-points improved between 2002 and 2017 (Supplementary Tables 6 and 7), the in-hospital and 1-year mortality rates by 2017 remained substantial (male: 15.7% and 32.2%; female: 21.6% and 37.5%, respectively).

### 4.1 Trends in adjusted all-cause mortality

**Fig. 2:**
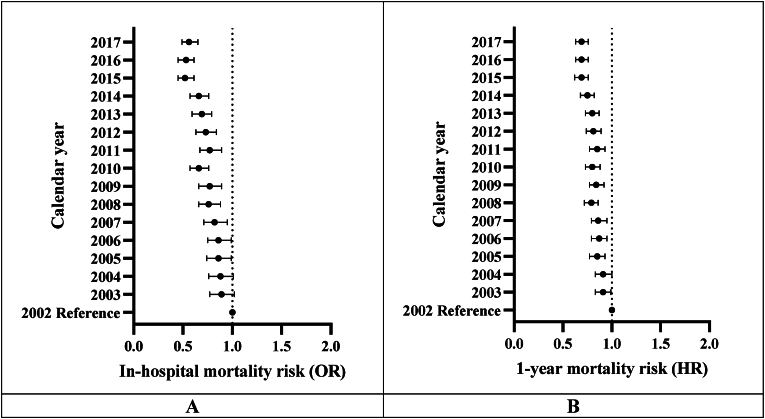
Forest plots of adjusted in-hospital and 1-year mortality stratified by calendar-year. Figure shows the forest plots of adjusted in-hospital mortality odds ratio (Panel A) and 1-year mortality hazard ratio (Panel B), with 2002 as the reference calendar year (see Supplementary Tables 10 and 11 respectively for full multivariable analyses results). HR, hazard ratio; OR, odds ratio.

To assess temporal trends in all-cause in-hospital and 1-year mortality following HS, multivariable analyses were performed to adjust for patient's age, sex, referral source, comorbidities, and calendar year of admission (see Supplementary Tables 8 and 9 for univariable predictors of in-hospital and 1-year mortality respectively). There was consistent improvement in outcomes during the study period: the adjusted odds ratio (aOR) for in-hospital mortality and adjusted hazard ratio (aHR) for 1-year mortality post-HS comparing patients admitted in 2017 to 2002 were 0.55 (95% confidence interval [CI] = 0.47–0.64) and 0.69 (95% CI = 0.63–0.76) (both p < 0.001), respectively (Fig. 2, Supplementary Tables 10 and 11).

Male sex was associated with a lower risk for in-hospital mortality (aOR = 0.83, 95% CI = 0.79–0.88, p < 0.001) and at 1-year (aHR = 0.93, 95% CI = 0.89–0.96, p < 0.001) (Table 2). For each 1-year increase in age, in-hospital and 1-year mortality increased by 4% (aOR = 1.04, 95% CI = 1.03–1.04, p < 0.001) and 3% (aHR = 1.03, 95% CI = 1.03–1.04, p < 0.001), respectively (Table 2). Comorbidity burden also independently predicted worse outcomes such that for each 1-point increase in CCI, in hospital and 1-year mortality increased by 6% (aOR = 1.06, 95% CI = 1.05–1.08, p < 0.001) and 10% (aHR = 1.10, 95% CI = 1.09–1.11, p = 0.002), respectively (Table 2). Surgical evacuation of ICH was associated with better mortality outcomes both in-hospital (aOR = 0.46, 95% CI = 0.41–0.52, p < 0.001) and at 1-year (aHR = 0.49, 95% CI = 0.45–0.53, p < 0.001) (Table 2). Hypertension was a significant independent predictor of better outcome at 1-year (aHR = 0.86, 95% CI = 0.83–0.89, p < 0.001) but not for in-hospital mortality (Table 2). Interestingly, hypertension was associated with increased 1-year mortality (HR = 1.08, 95% CI = 1.05–1.12, p < 0.001) in univariable analysis suggesting potentially an interaction term to account for this discrepancy. As an additional exploratory analysis, we identified a strong interaction between hypertension and age (p < 0.001 for interaction). When we stratified the study cohort by mean age (69.1yo), hypertension was associated with increased risk of 1-year mortality in univariable analysis but was not a significant independent predictor in multivariable analysis in those ≤69.1yo (Supplementary Table 12). In contrast, in patients >69.1yo, hypertension was associated with a lowered risk of 1-year all-cause mortality in both univariable and multivariable analyses. In addition, a history of hyperlipidaemia and current smoker status were associated with lower in-hospital and 1-year mortality (Table 2).

**Table 2:** Independent predictors for all-cause mortality^a^.

Parameters	In-hospital		1-year	
	OR (95% CI)	p-value	HR (95% CI)	p-value
Age – per-1yr	1.04 (1.03–1.04)	<0.001	1.03 (1.03–1.04)	<0.001
Male	0.83 (0.79–0.88)	<0.001	0.93 (0.89–0.96)	<0.001
Surgical evacuation of ICH	0.46 (0.41–0.52)	<0.001	0.49 (0.45–0.53)	<0.001
Referral source		<0.001		<0.001
Emergency Department	1.00 (reference)		1.00 (reference)	
Physician-referred	0.35 (0.29–0.42)	<0.001	0.51 (0.46–0.57)	<0.001
External hospital-referred	0.83 (0.75–0.91)	<0.001	0.90 (0.85–0.96)	0.001
Others	1.51 (1.13–2.01)	0.005	1.21 (1.02–1.45)	0.03
Unknown	1.00 (0.50–2.02)	1.00	0.87 (0.51–1.47)	0.60
Hypertension			0.86 (0.83–0.89)	<0.001
Hyperlipidaemia	0.56 (0.49–0.64)	<0.001	0.60 (0.54–0.65)	<0.001
Current smoker	0.82 (0.74–0.92)	<0.001	0.89 (0.83–0.96)	<0.001
CCI score – per 1-score^b^	1.06 (1.05–1.08)	<0.001	1.10 (1.09–1.11)	0.002
Year of admission		<0.001		<0.001
2002	1.00 (reference)		1.00 (reference)	
2003	0.88 (0.77–1.01)	0.08	0.91 (0.83–0.99)	0.04
2004	0.87 (0.75–1.00)	0.05	0.91 (0.83–1.00)	0.05
2005	0.85 (0.73–0.98)	0.02	0.85 (0.77–0.93)	0.001
2006	0.86 (0.74–0.99)	0.03	0.87 (0.79–0.95)	0.002
2007	0.81 (0.70–0.94)	0.004	0.86 (0.79–0.95)	0.002
2008	0.75 (0.65–0.87)	<0.001	0.79 (0.72–0.86)	<0.001
2009	0.76 (0.66–0.87)	<0.001	0.84 (0.77–0.92)	<0.001
2010	0.66 (0.57–0.76)	<0.001	0.80 (0.73–0.88)	<0.001
2011	0.76 (0.66–0.87)	<0.001	0.85 (0.77–0.93)	<0.001
2012	0.73 (0.63–0.84)	<0.001	0.81 (0.74–0.89)	<0.001
2013	0.68 (0.59–0.78)	<0.001	0.80 (0.73–0.87)	<0.001
2014	0.64 (0.56–0.75)	<0.001	0.75 (0.68–0.82)	<0.001
2015	0.52 (0.45–0.60)	<0.001	0.69 (0.62–0.76)	<0.001
2016	0.51 (0.44–0.59)	<0.001	0.69 (0.63–0.76)	<0.001
2017	0.55 (0.47–0.64)	<0.001	0.69 (0.63–0.76)	<0.001

CCI, Charlson comorbidity index; CI, confidence interval; HR, hazards ratio; ICH, intracranial haemorrhage; OR, odds ratio.

a Only variables with P < 0.05 in multivariable analysis are shown (see Supplementary Tables 10 and 11 for full multivariable analysis results).

b Conditions included in the Charlson Comorbidity Index include myocardial infarction, congestive cardiac failure, peripheral vascular disease, stroke, dementia, chronic pulmonary disease, connective tissue disease, peptic ulcer disease, liver disease (mild vs. moderate to severe), diabetes (with or without organ damage), hemiplegia, moderate to severe renal disease, any tumor (within last 5 years), lymphoma, leukemia, metastatic solid tumor and acquired immunodeficiency syndrome.

## 5 Discussion

The main salient findings from this large population-level study are as follows: 1) admission rates for acute HS have decreased significantly in both sexes between 2002 and 2017, with most of the decline being driven by patients above 60yo; 2) overall in-hospital and 1-year mortality post-HS have fallen by 45% and 31% respectively during the study period after adjusting for age, sex, referral source and comorbidities; 3) compared to males, females had significant worse in-hospital and 1-year mortality outcomes; 4) patients who underwent surgical evacuation of ICH had significantly better outcomes.

Compared to previous epidemiological studies on HS in Australia, our study is the largest to date and of longest duration allowing comprehensive temporal trend analyses over 16 years in a statewide population cohort. Gattellari et al. reported the average age-adjusted admission rates for ICH and SAH were 16 and 7 per 100,000-persons-per-annum respectively, in NSW Australia between 2005 and 2013 [3]. In contrast, our study reported much higher overall averaged sex-stratified HS admission rates between 2002 and 2017. The differences could be accounted for by methodological differences in deriving the adjusted admission rates. Our study had similar absolute case numbers to that reported by Gattellari et al., but admission rates were standardised to the NSW population by specific sex and age-groups rather than the world population providing a more accurate epidemiological estimation of HS rates. The incidence rates of HS vary significantly throughout the world; similar rates to our study have been reported in China [16] and Germany [10]. Age-adjusted HS admission rates were higher in males than females in our study (males 63.63 and females 49.93 admissions-per-100,000-persons-per-annum), consistent with other studies [11,17,18].

Other epidemiological studies in NSW found ICH admission rates declined significantly across 2001 to 2014 [3,9] while remaining stable for SAH from 2001 to 2009 [8]. Downward temporal trends in HS rates have also been observed in several other countries [[19], [20], [21], [22]]. Overall, global HS incidence was reported to have decreased by 19% in high-income countries from 1990 to 2010 [7]. Our study found that declining HS hospitalisation rates were predominantly driven by those above 60yo. Other international [10,23] studies also showed HS admission rates to decrease in patients above 70yo. While the decreased HS incidence in elderly patients is reassuring, HS burden of disease remain high and ongoing treatment and preventative efforts remain a priority.

Compared with reported HS cohorts from other Western countries [10,19,21,24], our cohort had a similar median age and gender distribution. Cardiovascular disease, hypertension, diabetes and smoking were the most commonly observed comorbidities within our cohort, similar to other studies [10,21]. Our study is one of few [[10], [11], [12]] which adjusted for comorbidities in the analysis of post-HS mortality outcomes. Multivariable analysis revealed a 45% decline in in-hospital mortality and a 31% decline in 1-year mortality post-HS from 2002 to 2017 in NSW. Similar trends have been reported in other countries [4,[25], [26], [27]]. Global studies have overall reported a 38% reduction in HS mortality rates in high-income countries between 1990 and 2010 [7]. The mechanisms for this trend could be partly explained by results from The National Stroke Audit in Australia in 2019, which found an increased use of rapid triage and stroke protocols in emergency departments along with increasingly rapid access to brain imaging for diagnosis [28]. In addition, the proportion of hospitals with stroke units has increased from 26% to 79% between 1999 and 2017 [28], with access to stroke units shown to be associated with greatly improved patient outcomes [29]. Finally, improvements in the provision of patient advice for stroke risk factor modification could be another driver in improving mortality outcomes [28]. An Australian study reported that following ICH, patients with hypertension represented 89% of those prescribed with anti-hypertensive medications on discharge [30]. Further prospective studies are required to fully elucidate the effects of these interventions on HS mortality rates.

Significantly higher crude cumulative mortality rates at all time points post-HS were observed in females compared to males in our study. Multivariable analysis found that females had a 12% greater risk of in-hospital mortality post-HS compared to males. A study in Melbourne, Australia, also reported a significantly higher HS case fatality in females [18]. HS mortality has also been found to be greater in females in other countries [10,11]. This phenomenon has previously been attributed to factors such as differing rates of comorbid conditions and oral anti-coagulant use between the sexes, increased risk for misdiagnosis on stroke presentation in women, and increased cerebrovascular risks associated with infertility and adverse pregnancy outcomes [[31], [32], [33]]. Another possible driver could be due to differences in rates of interventions. We observed more males underwent surgical evacuation for ICH compared to females (13.3% vs 7.3%). A study from Spain reported higher in-hospital mortality post-HS in females who also had lower rates of decompressive craniectomy compared to males [11]. The exact drivers behind women having increased in-hospital mortality post-HS remain unclear and require further elucidation to bridge this gender outcome disparity.

In the present study, increasing age was found to be a predictor of increased in-hospital and 1-year mortality risk post-HS independent of comorbidities. Another study found age ≥65 to be an independent predictor for 30-day mortality in ICH patients [12]. Possible drivers include pre-stroke disability and stroke severity which are shown to be greater in the elderly [34,35]. Another independent predictor of increased mortality post-HS was increased comorbidity burden. For each 1-point increase in CCI, in-hospital and 1-year mortality risk increased by 6% and 10% respectively. Other studies found CCI to be an independent predictor of length of stay following HS [36] and increased in-hospital mortality following HS [37]. Management and prevention of stroke may be more complicated in patients with multiple comorbidities due to increased risk of adverse clinical and pharmacological interactions. One study reported patients with higher CCI scores were less likely prescribed anti-hypertensive therapy following a stroke [30].

The benefit of surgical evacuation in ICH patients remains a controversial topic. The present study found surgical evacuation was associated with improved mortality outcomes both in-hospital and at 1-year. De Miguel-Yanes et al. similarly showed decompressive craniectomy was associated with increased survival of 51% in females and 57% in males following hospital discharge post-HS [11]. This is contrary to findings from a German study which found surgical evacuation of ICH was associated with an 86% increase in in-hospital mortality [10]. The decision to undergo surgery in ICH patients is complicated by a multitude of factors including the location of the haematoma, and the type of surgery [38,39]. Moreover, our study could not account for possible survival bias such that only patients clinically deemed most likely to survive and thus ‘worthwhile’ to salvage following an acute HS event are offered surgery. In the current clinical guidelines, recommendations for surgical evacuation of ICH remain weak due to uncertainties in the current literature with some exceptions for patients with highly-specific clinical indications [40]. The therapeutic efficacy of surgical evacuation for ICH remains an area for further research. The EVACUATE study is an up-and-coming randomised controlled trial conducted in Melbourne, Australia, which will assess ultra-early, minimally invasive, haematoma evacuation in comparison to standard care in ICH patients (ClinicalTrials.gov Identifier: NCT04434807).

An unexpected finding in the present study was that patients with history of current smoking had improved mortality outcomes both in-hospital and at 1-year after adjusting for age, sex and other comorbidities. The association between smoking and cardiovascular disease remains inconsistent in the current literature. Some studies have reported significant increased risk of HS mortality in smokers compared to non-smokers [41,42], while other studies found no significant difference [43,44]. One study showed improved mortality and functional outcomes post-stroke in smokers compared to non-smokers, although these differences became insignificant after matching for age and sex [45]. Current stroke guidelines recommend smoking cessation for patients post-HS [39]. As our study is retrospective in nature, the cause-effect relationship between smoking and improved mortality in HS patients cannot be established and remains an interesting albeit surprising observation that requires further exploration. Interestingly, our study found differential univariable and multivariable results on the impact of hypertension on 1-year all-cause mortality post-acute HS which appears to be a consequence of strong interaction with age. We intend to examine this relationship in detail in a future study. In addition, we found a history of hyperlipidaemia to be an independent predictor of favourable mortality outcomes. Current literature has reported an inverse relationship between serum cholesterol levels and risk for HS [[46], [47], [48]]. In ICH patients, low serum low density lipoprotein cholesterol level was found to be an independent predictor for haematoma growth, early neurological deterioration and 3-month mortality [49]. Lipid-lowering medications, particularly statins, have also been linked to increased HS risk [47]. Nevertheless, there remains a degree of uncertainty in these conclusions [50] and further work is required to validate our study's finding.

### 5.1 Limitations

This study is a retrospective population-linkage cohort study and is limited by its observational nature. Thus, mechanistic explanations for our observations can only be inferred and exact causative relationships cannot be drawn. While we have adjusted for many variables, unaccounted confounding factors may remain in our dataset which could lead to differences in our study outcomes compared to others. Our study design is dependent on accurate data coding, hence potential error or changes in ICD-10AM coding protocols in hospital systems may skew our observations though we were unaware of any substantial changes to the ICD-10AM codings during our study period. Furthermore, our analyses were limited to index HS cases and cannot account for the impact of repeated HS presentations. Our study findings are only applicable to patients who were admitted for acute HS and cannot account for patients who were not admitted or died prior to admission. Thus, our observed hospital admission rates may not reflect the true incidence of HS in NSW. At the very least, our study provides an estimation that can be used for reference in future studies. Due to the administrative nature of our data, clinical factors that may be relevant to patient outcomes were not available including stroke severity, pharmacological and medical interventions, and post-HS outcomes. Finally, though subtypes of HS have been reported to have different outcomes in a previous study [3], we did not separate HS into its various subtypes to minimize the potential of underestimating the rates of HS.

## 6 Conclusions

In this large Australian statewide cohort study, overall age-adjusted admission rates have fallen from 2002 to 2017 for both sexes, with the decline driven mostly by 60yo and older age-groups. Though acute and medium-term mortality following HS had improved significantly, more than one in six patients still died in-hospital and one in three patients failed to survive past one year in the study period. Surgical evacuation following HS and a history of current smoking and hyperlipidaemia were associated with decreased mortality risk, while female sex, increasing age and increased comorbidity burden independently predicted worse outcomes. Future research is required to mitigate this highly lethal cardiovascular disease particularly in at-risk populations.

## Funding

Nil.

## CRediT authorship contribution statement

**Arielle Chin-yu Hsu:** Writing – review & editing, Writing – original draft, Methodology, Investigation, Formal analysis. **Vijayatubini Vijayarajan:** Writing – review & editing. **Yeu-Yao Cheng:** Writing – review & editing. **Matthew Wei Shun Shu:** Writing – review & editing. **Karice Hyun:** Writing – review & editing. **Vincent Chow:** Writing – review & editing. **David Brieger:** Writing – review & editing. **Leonard Kritharides:** Writing – review & editing. **Austin Chin Chwan Ng:** Writing – review & editing, Writing – original draft, Visualization, Validation, Supervision, Resources, Project administration, Methodology, Investigation, Formal analysis, Data curation, Conceptualization.

## Declaration of competing interest

The authors declare that they have no known competing financial interests or personal relationships that could have appeared to influence the work reported in this paper.
