# Supplementary material for: Mortality outcomes in 35,433 patients admitted for acute haemorrhagic stroke in Australia: A population-linkage study
Source: Int J Cardiol Cardiovasc Risk Prev. 2024 Mar 20;21:200258. doi: 10.1016/j.ijcrp.2024.200258 (PMC10972823; doi:10.1016/j.ijcrp.2024.200258)
Supplement: Multimedia component 1 [file mmc1.docx]

**SUPPLEMENTARY MATERIAL**

**Mortality outcomes in 35433 patients admitted for acute haemorrhagic stroke in Australia: a population-linkage study**

**Hsu et al.**

**Table of Contents**

**Supplementary Table 1. Study comorbidities International Classification of Diseases Tenth Revision Australian Modification (ICD-10AM) codes and Australian Classification of Health Interventions (ACHI) procedural codes.**

**Supplementary Table 2. Volume and age-adjusted admission rates of haemorrhagic stroke by sex and calendar year.**

**Supplementary Table 3. Female age-specific haemorrhagic stroke admission rates stratified by calendar year.**

**Supplementary Table 4. Male age-specific haemorrhagic stroke admission rates stratified by calendar year.**

**Supplementary Table 5. Cumulative mortality post-haemorrhagic stroke for females.**

**Supplementary Table 6. Cumulative mortality post-haemorrhagic stroke for males.**

**Supplementary Table 7. Univariable predictors for in-hospital all-cause mortality.**

**Supplementary Table 8. Univariable predictors for 1-year all-cause mortality.**

**Supplementary Table 9. Multivariable predictors for in-hospital all-cause mortality.**

**Supplementary Table 10. Multivariable predictors for 1-year all-cause mortality.**

**Supplementary Figure 1. Derivation of study cohort.**

**Supplementary Figure 2. Kaplan-Meier survival curve following admission for haemorrhagic stroke.**

**Supplementary Table 1. Study comorbidities International Classification of Diseases, Tenth Revision Australian Modification (ICD-10AM) codes and Australian Classification of Health Interventions (ACHI) procedural codes.**

| **No.** | **Comorbidity *** | **ICD-10AM codes** |
| --- | --- | --- |
| 1 | Pulmonary embolism | I26, I26.0, I26.9 |
| 2 | Atrial fibrillation and/or flutter | I48, I48.0, I48.1, I48.2, I48.3, I48.4, I48.9 |
| 3 | Acute myocardial infarction | I21, I21.0, I21.1, I21.2, I21.3, I21.4, I21.9, I22, I22.0, I22.1, I22.8, I22.9 |
| 4 | Ischaemic heart disease | I20, I20.0, I20.1, I20.8, I20.9, I21, I21.0, I21.1, I21.2, I21.3, I21.4, I21.9, I22, I22.0, I22.1, I22.8, I22.9, I23, I23.0, I23.1, I23.2, I23.3, I23.4, I23.5, I23.6, I23.8, I24, I24.0, I24.1, I24.8, I24.9, I25, I25.0, I25.1, I25.10, I25.11, I25.12, I25.13, I25.2, I25.3, I25.4, I25.5, I25.6, I25.8, I25.9 |
| 5 | Prior percutaneous coronary intervention (PCI) / coronary artery bypass graft (CABG) | Z95.1, Z95.5 |
| 6 | Congestive cardiac failure | I42, I42.0, I42.1, I42.2, I42.3, I42.4, I42.5, I42.6, I42.7, I42.8, I42.9, I43, I43.0, I43.1, I43.2, I43.8, I50, I50.0, I50.1, I50.9, I11.0, I13.0, I13.2 |
| 7 | Valvular heart disease | I05, I05.0, I05.1, I05.2, I05.8, I05.9, I06, I06.0, I06.1, I06.2, I06.8, I06.9, I07, I07.0, I07.1, I07.2, I07.8, I07.9, I08, I08.0, I08.1, I08.2, I08.3, I08.8, I08.9, I09.1, I09.8, I34, I34.0, I34.1, I34.2, I34.8, I34.9, I35, I35.0, I35.1, I35.2, I35.8, I35.9, I36, I36.0, I36.1, I36.2, I36.8, I36.9, I37, 37.0, I37.1, I37.2, I37.8, I37.9, I38, I39, I39.0, I39.1, I39.2, I39.3, I39.4, I39.8, Q22, Q22.0, Q22.1, Q22.2, Q22.3, Q22.30, Q22.31, Q22.39, Q22.4, Q22.41, Q22.42, Q22.5, Q22.6, Q22.8, Q22.81, Q22.82, Q22.89, Q22.9, Q23, Q23.0, Q23.01, Q23.02, Q23.1, Q23.2, Q23.21, Q23.22, Q23.3, Q23.4, Q23.8, Q23.81, Q23.82, Q23.83, Q23.89, Q23.9, Q24.87 |
| 8 | Peripheral vascular disease | E09.5, E09.51, E09.52, E10.51, E10.52, E11.51, E11.52, E13.51, E13.52, E14.51, E14.52, I70, I70.0, I70.1, I70.2, I70.20, I70.21, I70.22, I70.23, I70.24, I70.8, I70.9, I71, I71.0, I71.00, I71.01, I71.02, I71.03, I71.1, I71.2, I71.3, I71.4, I71.5, I71.6, I71.8, I71.9, I72, I72.0 I72.1, I72.2, I72.3, I72.4, I72.5, I72.6, I72.8, I72.9, I73, I73.0, I73.1, I73.8, I73.9, I74, I74.0, I74.1, I74.2, I74.3, I74.4, I74.5, I74.8, I74.9, I77, I77.0, I77.1, I77.2, I77.3, I77.4, I77.5, I77.6, I77.8, I77.9, I78, I78.0, I78.1, I78.8, I78.9, I79, I79.0, I79.1, I79.2, I79.8 |
| 9 | Stroke | Haemorrhage: I60, I60.0, I60.1, I60.2, I60.3, I60.4, I60.5, I60.6, I60.7, I60.8, I60.9, I61, I61.0, I61.1, I61.2, I61.3, I61.4, I61.5, I61.6, I61.8, I61.9, I62, I62.0, I62.1, I62.9  Infarction: I63, I63.0, I63.1, I63.2, I63.3, I63.4, I63.5, I63.6, I63.8, I63.9, I64  Transient ischaemic attack: G45, G45.0, G45.1, G45.2, G45.3, G45.4, G45.8, G45.9 |
| 10 | Prosthetic heart valve | Z95.2, Z95.3, Z95.4 |
| 11 | Cardiovascular disease | Defined as morbidities item nos. 4, 6, 9, 10, 11, 12, 13, 18 |
| 12 | Hypertension | I10, I11, I11.0, I11.9, I12, I12.0, I12.9, I13, I13.0, I13.1, I13.2, I13.9, I15, I15.0, I15.1, I15.2, I15.8, I15.9 |
| 13 | Hyperlipidaemia | E78, E78.0, E78.1, E78.2, E78.3, E78.4, E78.5, E78.6, E78.8, E78.9 |
| 14 | Diabetes | E09, E09.2, E09.21, E09.29, E09.3, E09.31, E09.32, E09.4, E09.40, E09.42, E09.5, E09.51, E09.52, E09.7, E09.71, E09.72, E09.8, E09.9, E10, E10.0, E10.01, E10.02, E10.1, E10.11, E10.12, E10.13, E10.14, E10.15, E10.16, E10.2, E10.21, E10.22, E10.29, E10.3, E10.31, E10.32, E10.33, E10.34, E10.35, E10.36, E10.39, E10.4, E10.40, E10.41, E10.42, E10.43, E10.49, E10.5, E10.51, E10.52, E10.53, E10.6, E10.61, E10.62, E10.63, E10.64, E10.65, E10.69, E10.7, E10.71, E10.73, E10.8, E10.9, E11, E11.0, E11.01, E11.02, E11.1, E11.11, E11.12, E11.13, E11.14, E11.15, E11.16, E11.2, E11.21, E11.22, E11.29, E11.3, E11.31, E11.32, E11.33, E11.34, E11.35, E11.36, E11.39, E11.4, E11.40, E11.41, E11.42, E11.43, E11.49, E11.5, E11.51, E11.52, E11.53, E11.6, E11.61, E11.62, E11.63, E11.64, E11.65, E11.69, E11.7, E11.71, E11.72, E11.73, E11.8, E11.9, E13, E13.0, E13.01, E13.02, E13.1, E13.11, E13.12, E13.13, E13.14, E13.15, E13.16, E13.2, E13.21, E13.22, E13.29, E13.3, E13.31, E13.32, E13.33, E13.34, E13.35, E13.36, E13.39, E13.4, E13.40, E13.41, E13.42, E13.43, E13.49, E13.5, E13.51, E13.52, E13.53, E13.6, E13.61, E13.62, E13.63, E13.64, E13.65, E13.69, E13.7, E13.71, E13.72, E13.73, E13.8, E13.9, E14, E14.0, E14.01, E14.02, E14.1, E14.11, E14.12, E14.13, E14.14, E14.15, E14.16, E14.2, E14.21, E14.22, E14.29, E14.3, E14.31, E14.32, E14.33, E14.34, E14.35, E14.36, E14.39, E14.4, E14.40, E14.41, E14.42, E14.43, E14.49, E14.5, E14.51, E14.52, E14.53, E14.6, E14.61, E14.62, E14.63, E14.64, E14.65, E14.69, E14.7, E14.71, E14.72, E14.73, E14.8, E14.9, Z92.22 |
| 15 | Current smoker | F17, Z72.0 |
| 16 | Systemic connective tissue disease | M30, M30.0, M30.1, M30.2, M30.3, M30.8, M31, M31.0, M31.1, M31.2, M31.3, M31.4, M31.5, M31.6, M31.7, M31.8, M31.9, M32, M32.0, M32.1, M32.8, M32.9, M33, M33.0, M33.1, M33.2. M33.9, M34, M34.0, M34.1, M34.2, M34.8, M34.9, M35, M35.0, M35.1, M35.2, M35.3, M35.4, M35.5, M35.6, M35.7, M35.8, M35.9, M36, M36.0, M36.1, M36.2, M36.3, M36.4, M36.8 |
| 17 | Chronic pulmonary disease | E84.0, E84.0, E84.1, E84.8, E84.9, J40, J41, J41.0, J41.1, J41.8, J42, J43, J43.0, J43.1, J43.2, J43.8, J43.9, J44, J44.0, J44.1, J44.8, J44.9, J45, J45.0, J45.1, J45.8, J45.9, J46, J47, J60, J61, J62, J62.0, J62.8, J63, J63.0, J63.1, J63.2, J63.3, J63.4, J63.5, J63.8, J64, J65, J66, J66.0, J66.1, J66.2, J66.8, J67, J67.0, J67.1, J67.2, J67.3, J67.4, J67.5, J67.6, J67.7, J67.8, J67.9, J68, J68.0, J68.1, J68.2, J68.3, J68.4, J68.8, J68.9, J70, J70.0, J70.1, J70.2, J70.3, J70.4, J70.8, J70.9, J82, J84, J84.0, J84.1, J84.8. J84.9, J99, J99.1, J99.8 |
| 18 | Malignancy | C00-C96, D00-D09 |
| 19 | Chronic kidney disease | N18, N18.1, N18.2, N18.3, N18.4, N18.5, N18.9, N19 |
| 20 | Dementia | F00, F00.0, F00.1, F00.2, F00.9, F01, F01.0, F01.1, F01.2, F01.3, F01.8, F01.9, F02, F02.0, F02.1, F02.2, F02.3, F02.4, F02.8, F03 |
| 21 | Neurodegenerative diseases (defined as dementia, central nervous systemic atrophies, Parkinson’s disease, basal ganglia degeneration and/or nervous systemic degenerative diseases) | F00, F00.0, F00.1, F00.2, F00.9, F01, F01.0, F01.1, F01.2, F01.3, F01.8, F01.9, F02, F02.0, F02.1, F02.2, F02.3, F02.4, F02.8, F03, G10, G11, G11.0, G11.1, G11.2, G11.3, G11.4, G11.8, G11.9, G12, G12.0, G12.1, G12.2, G12.8, G12.9, G13, G13.0, G13.1, G13.2, G13.8, G14, G20, G23, G23.0, G23.1, G23.2, G23.8, G23.9, G30, G30.0, G30.1, G30.8, G30.9, G31, G31.0, G31.1, G31.2, G31.3, G31.8, G31.9 |
| 22 | Peptic ulcer disease | K25, K26, K27, K28 |
| 23 | Liver disease – mild | K70.0, K70.1, K70.2, K70.9, K71.0, K71.1, K71.2, K71.3, K71.4, K71.5, K71.6, K71.8, K71.9, K73, K73.0, K73.1, K73.2, K73.8, K73.9, K75, K75.0, K75.1, K75.2, K75.3, K75.4, K75.8, K75.9, K76, K76.0, K76.1, K76.2, K76.3, K76.4, K76.5, K76.6, K76.7, K76.8, K76.9, K77, K77.0, K77.8 |
| 24 | Liver disease – mod-severe | I82.0, K70.3, K70.4, K71.7, K72, K72.0, K72.1, K72.9, K74, K74.0, K74.1, K74.2, K74.3, K74.4, K74.5, K74.6 |
| 25 | Chronic kidney disease – mod-severe | N18.3, N18.4, N18.5 |
| 26 | Diabetes with organ damage | E09.21, E09.29, E09.31, E09.32, E09.40, E09.42, E09.51, E09.52, E09.71, E09.72, E09.8, E10.21, E10.22, E10.29, E10.31, E10.32, E10.33, E10.34, E10.35, E10.36, E10.39, E10.40, E10.41, E10.42, E10.43, E10.49, E10.51, E10.52, E10.53, E10.61, E10.62, E10.63, E10.69, E10.71, E10.73, E10.8, E11.21, E11.22, E11.29, E11.31, E11.32, E11.33, E11.34, E11.35, E11.36, E11.39, E11.40, E11.41, E11.42, E11.43, E11.49, E11.51, E11.52, E11.53, E11.61, E11.62, E11.63, E11.69, E11.71, E11.72, E11.73, E11.8, E13.21, E13.22, E13.29, E13.31, E13.32, E13.33, E13.34, E13.35, E13.36, E13.39, E13.40, E13.41, E13.42, E13.43, E13.49, E13.51, E13.52, E13.53, E13.61, E13.62, E13.63, E13.69, E13.71, E13.72, E13.73, E13.8, E14.21, E14.22, E14.29, E14.31, E14.32, E14.33, E14.34, E14.35, E14.36, E14.39, E14.40, E14.41, E14.42, E14.43, E14.49, E14.51, E14.52, E14.53, E14.61, E14.62, E14.63, E14.69, E14.71, E14.72, E14.73, E14.8 |
| 27 | Lymphoma | C81, C81.0, C81.1, C81.2, C81.3, C81.4, C81.7, C81.9, C82, C82.0, C82.1, C82.2, C82.3, C82.4, C82.5, C82.6, C82.7, C82.9, C83, C83.0, C83.1, C83.3, C83.5, C83.7, C83.8, C83.9, C84, C84.0, C84.1, C84.4, C84.5, C84.6, C84.7, C84.8, C84.9, C85, C85.1, C85.2, C85.7, C85.9, C86, C86.0, C86.1, C86.2, C86.3, C86.4, C86.5, C86.6, C88, C88.0, C88.2, C88.3, C88.4, C88.7, C88.9 |
| 28 | Leukaemia | C90, C90.0, C90.1, C90.2, C90.3, C91, C91.0, C91.1, C91.3, C91.4, C91.5, C91.6, C91.7, C91.8, C91.9, C92, C92.0, C92.1, C92.2, C92.3, C92.4, C92.5, C92.6, C92.7, C92.8, C92.9, C93, C93.0, C93.1, C93.3, C93.7, C93.9, C94, C94.0, C94.2, C94.3, C94.4, C94.6, C94.7, C95, C95.0, C95.1, C95.7, C95.9, C96, C96.0, C96.2, C96.4, C96.5, C96.6, C96.7, C96.8, C96.9 |
| 29 | Metastatic solid tumour | C76, C76.0, C76.1, C76.2, C76.3, C76.4, C76.5, C76.7, C76.8, C77, C77.0, C77.1, C77.2, C77.3, C77.4, C77.5, C77.8, C77.9, C78, C78.0, C78.1, C78.2, C78.3, C78.4, C78.5, C78.6, C78.7, C78.8, C79, C79.0, C79.1, C79.2, C79.3, C79.4, C79.5, C79.6, C79.7, C79.8, C79.81, C79.82, C79.88, C79.9, C80, C80.0, C80.9 |
| 30 | AIDS | B20, B21, B22, B23, B23.0, B23.8, B24 |
| 31 | Hemiplegia | G81, G81.0, G81.1, G81.9, G82, G82.0, G82.1, G82.2, G82.3, G82.4, G82.5 |
| 32 | Any tumour/malignancy excluding lymphoma and/or leukemia | C00-C80, D00-D09 |

- To calculate the Charlson comorbidity index (CCI) score, without age adjustment, for individual patient during a particular admission of interest, use the following morbidity item numbers with their corresponding ICD-10AM codes to derive the particular patient’s CCI score:
  1. 1 score for each morbidity item – 3, 6, 8, 9, 14, 16, 17, 20, 22, 23
  2. 2 score for each morbidity item – 25, 26, 27, 28, 31, 32
  3. 3 score for morbidity item – 24
  4. 6 score for morbidity item – 29, 30

| **No.** | **ACHI procedures** | **ACHI procedural codes** |
| --- | --- | --- |
| 1 | Surgical evacuation of intracranial haemorrhage | 39009-00, 39600-00, 39603-00, 39603-01 |

**Supplementary Table 2. Volume and age-adjusted admission rates of haemorrhagic stroke by sex and calendar year.**

| **Calendar year** | **Admissions** | | **NSW population*** | | **Age-adjusted admission rates†** | |
| --- | --- | --- | --- | --- | --- | --- |
|  | **Females** | **Males** | **Female** | **Male** | **Female** | **Male** |
| 2002 | 1,053 | 1,169 | 3,315,023 | 3,265,784 | 55.20 | 74.39 |
| 2003 | 1,131 | 1,058 | 3,337,006 | 3,283,709 | 58.33 | 69.02 |
| 2004 | 1,053 | 1,070 | 3,353,338 | 3,297,397 | 53.10 | 68.32 |
| 2005 | 1,010 | 1,049 | 3,376,712 | 3,316,494 | 51.24 | 68.23 |
| 2006 | 1,109 | 1,165 | 3,403,655 | 3,339,035 | 55.98 | 71.96 |
| 2007 | 1,038 | 1,111 | 3,447,018 | 3,387,138 | 50.54 | 65.01 |
| 2008 | 1,109 | 1,125 | 3,498,364 | 3,445,097 | 52.03 | 68.08 |
| 2009 | 1,067 | 1,130 | 3,550,819 | 3,502,936 | 50.12 | 63.55 |
| 2010 | 1,069 | 1,146 | 3,596,185 | 3,548,107 | 48.79 | 63.26 |
| 2011 | 1,106 | 1,096 | 3,633,420 | 3,585,109 | 49.95 | 60.36 |
| 2012 | 1,109 | 1,126 | 3,677,844 | 3,626,400 | 48.37 | 59.25 |
| 2013 | 1,058 | 1,211 | 3,729,622 | 3,674,410 | 45.59 | 63.80 |
| 2014 | 1,067 | 1,096 | 3,784,138 | 3,724,215 | 44.82 | 54.72 |
| 2015 | 1,070 | 1,167 | 3,839,594 | 3,776,574 | 43.83 | 57.18 |
| 2016 | 1,166 | 1,244 | 3,899,051 | 3,833,807 | 47.37 | 58.43 |
| 2017 | 1,108 | 1,147 | 3,966,912 | 3,901,024 | 43.64 | 52.54 |
| **Total** | 17,323 | 18,110 | − | − | − | − |
| **Mean±SD** | 1,083±39 | 1,132±53 | − | − | 49.93±4.35 | 63.63±6.22 |
|  |  |  |  |  |  |  |
| **Coefficient** | 2.90 | 5.74 | − | − | -0.83 | -1.20 |
| **R^2^** | 0.06 | 0.21 | − | − | 0.82 | 0.84 |
| **p-value** | 0.06 | 0.04 | − | − | <0.001 | <0.001 |
| NSW, New South Wales; SD, standard deviation.   - NSW population size for each calendar year was obtained from publicly available data from the Australian Bureau of Statistics (ABS). - Admission rates are presented as per-100,000-persons and adjusted for sex and age for each calendar year. For each patient, only their index hospital admission for a stroke was included. Admission rates are represented as per-100,000-person and adjusted for sex and age in decades (0-9, 10-19, 20-29, 30-39, 40-49, 50-59, 60-69, 70-79, >80 years) for each calendar year. The admission rates were calculated as the number of haemorrhagic stroke admissions divided by the sex and age group-specific NSW population size for each calendar year. Linear regression analysis was used to analyse temporal trends. | | | | | | |

**Supplementary Table 3. Female age-specific haemorrhagic stroke admission rates* stratified by calendar year.**

| **Calendar Year** | **Females Haemorrhagic Stroke Hospital Admission Rates (per 100,000) by Age Group** | | | | | | | | |
| --- | --- | --- | --- | --- | --- | --- | --- | --- | --- |
|  | **0-9** | **10-19** | **20-29** | **30-39** | **40-49** | **50-59** | **60-69** | **70-79** | **>80** |
| 2002 | 2.33 | 0.68 | 3.80 | 9.59 | 20.71 | 27.29 | 50.33 | 122.76 | 259.27 |
| 2003 | 1.64 | 2.05 | 4.47 | 6.80 | 20.45 | 31.29 | 56.13 | 137.30 | 264.85 |
| 2004 | 1.89 | 1.14 | 3.35 | 8.66 | 21.09 | 30.24 | 50.93 | 112.23 | 248.38 |
| 2005 | 1.19 | 1.82 | 3.32 | 8.88 | 19.39 | 26.79 | 42.73 | 95.77 | 261.38 |
| 2006 | 1.65 | 1.59 | 4.14 | 8.89 | 16.36 | 27.26 | 43.87 | 131.84 | 268.17 |
| 2007 | 0.23 | 1.81 | 3.21 | 8.84 | 18.37 | 27.03 | 42.99 | 104.62 | 247.73 |
| 2008 | 1.39 | 2.26 | 3.12 | 8.18 | 17.56 | 31.64 | 48.63 | 117.04 | 238.45 |
| 2009 | 1.36 | 2.03 | 3.84 | 6.54 | 16.09 | 26.77 | 45.11 | 102.66 | 246.65 |
| 2010 | 1.80 | 0.90 | 2.59 | 6.91 | 18.63 | 28.01 | 41.18 | 99.95 | 239.17 |
| 2011 | 1.34 | 1.36 | 2.18 | 5.53 | 15.71 | 30.40 | 42.05 | 105.62 | 245.33 |
| 2012 | 1.76 | 1.59 | 3.53 | 6.70 | 18.53 | 28.99 | 42.18 | 94.88 | 237.20 |
| 2013 | 1.51 | 1.59 | 2.71 | 7.21 | 16.87 | 29.14 | 34.59 | 90.44 | 226.23 |
| 2014 | 1.06 | 1.58 | 1.52 | 7.48 | 16.22 | 25.25 | 45.73 | 88.93 | 215.56 |
| 2015 | 1.04 | 2.26 | 2.80 | 6.58 | 16.73 | 27.31 | 42.38 | 87.58 | 207.79 |
| 2016 | 0.82 | 1.57 | 2.74 | 7.16 | 19.36 | 30.03 | 44.08 | 84.46 | 236.07 |
| 2017 | 1.63 | 1.54 | 2.68 | 6.77 | 15.96 | 27.91 | 43.73 | 84.66 | 207.83 |
| **Mean ± SD** | 1.42 ± 0.49 | 1.61 ± 0.44 | 3.17 ± 0.75 | 7.54 ± 1.14 | 18.00 ± 1.82 | 28.46 ± 1.84 | 44.79 ± 4.90 | 103.8 ± 16.5 | 240.6 ± 18.8 |
| **Coefficient** | -0.0364 | 0.0174 | -0.109 | -0.152 | -0.236 | -0.0481 | -0.611 | -2.89 | -3.44 |
| **R^2^** | 0.063 | -0.034 | 0.44 | 0.40 | 0.34 | -0.055 | 0.31 | 0.68 | 0.74 |
| **p-value** | 0.18 | 0.49 | 0.0031 | 0.0087 | 0.011 | 0.647 | 0.015 | <0.001 | <0.001 |

SD, standard deviation.

- Admission rates are represented as per-100,000-person. For each patient, only their index hospital admission for haemorrhagic stroke was included. Sex and age-specific admission rates were calculated as the number of haemorrhagic stroke admissions divided by the sex and age group-specific New South Wales (NSW) population size for each calendar year. NSW population data was obtained from publicly available data from the Australian Bureau of Statistics (ABS). Linear regression analysis was used to analyse temporal trends in admission rates.

**Supplementary Table 4. Male age-specific haemorrhagic stroke admission rates* stratified by calendar year.**

| **Calendar Year** | **Males Haemorrhagic Stroke Hospital Admission Rates (per 100,000) by Age Group** | | | | | | | | |
| --- | --- | --- | --- | --- | --- | --- | --- | --- | --- |
|  | **0-9** | **10-19** | **20-29** | **30-39** | **40-49** | **50-59** | **60-69** | **70-79** | **>80** |
| 2002 | 1.77 | 2.83 | 6.18 | 10.47 | 27.69 | 41.61 | 79.17 | 160.71 | 339.05 |
| 2003 | 2.23 | 3.68 | 3.74 | 8.30 | 17.92 | 28.48 | 67.69 | 183.49 | 305.60 |
| 2004 | 1.57 | 1.08 | 5.93 | 7.76 | 20.47 | 32.02 | 70.69 | 162.56 | 312.77 |
| 2005 | 1.80 | 1.73 | 4.79 | 5.94 | 15.96 | 32.81 | 70.01 | 146.48 | 334.58 |
| 2006 | 3.15 | 1.95 | 6.44 | 8.22 | 16.62 | 36.22 | 75.33 | 166.52 | 333.17 |
| 2007 | 2.66 | 3.01 | 3.35 | 9.63 | 19.71 | 35.73 | 63.51 | 155.32 | 292.17 |
| 2008 | 2.41 | 2.56 | 4.65 | 6.10 | 20.12 | 29.02 | 61.21 | 148.42 | 338.23 |
| 2009 | 3.23 | 2.77 | 4.11 | 7.65 | 20.49 | 31.52 | 64.31 | 140.48 | 297.37 |
| 2010 | 1.49 | 2.57 | 1.74 | 10.01 | 16.35 | 35.44 | 63.43 | 138.96 | 299.34 |
| 2011 | 1.48 | 2.14 | 3.48 | 7.79 | 17.68 | 27.47 | 53.19 | 136.61 | 293.38 |
| 2012 | 1.66 | 1.29 | 3.84 | 8.34 | 15.58 | 30.91 | 58.51 | 135.92 | 277.20 |
| 2013 | 2.85 | 1.29 | 3.60 | 9.22 | 18.98 | 30.73 | 57.47 | 129.42 | 320.60 |
| 2014 | 1.61 | 0.86 | 4.48 | 7.73 | 16.93 | 32.31 | 51.18 | 114.50 | 262.91 |
| 2015 | 2.57 | 1.92 | 3.29 | 7.39 | 16.47 | 31.53 | 53.88 | 128.81 | 268.77 |
| 2016 | 2.53 | 1.69 | 3.05 | 8.88 | 16.19 | 37.78 | 57.45 | 130.31 | 267.96 |
| 2017 | 2.32 | 2.49 | 3.84 | 4.50 | 17.43 | 30.22 | 52.57 | 120.68 | 238.80 |
| **Mean ± SD** | 2.21 ± 0.60 | 2.12 ± 0.78 | 4.16 ± 1.23 | 8.00 ± 1.55 | 18.41 ± 2.99 | 32.74 ± 3.74 | 62.48 ± 8.38 | 143.7 ± 18.3 | 298.9 ± 30.1 |
| **Coefficient** | 0.0144 | -0.0651 | -0.146 | -0.0912 | -0.334 | -0.181 | -1.56 | -3.45 | -5.03 |
| **R^2^** | -0.057 | 0.097 | 0.27 | 0.013 | 0.23 | -0.014 | 0.77 | 0.79 | 0.63 |
| **p-value** | 0.67 | 0.13 | 0.023 | 0.29 | 0.034 | 0.39 | <0.001 | <0.001 | <0.001 |

SD, standard deviation.

- Admission rates are represented as per-100,000-person. For each patient, only their index hospital admission for haemorrhagic stroke was included. Sex and age-specific admission rates were calculated as the number of haemorrhagic stroke admissions divided by the sex and age group-specific New South Wales (NSW) population size for each calendar year. NSW population data was obtained from publicly available data from the Australian Bureau of Statistics (ABS). Linear regression analysis was used to analyse temporal trends in admission rates.

**Supplementary Table 5. Cumulative mortality post-haemorrhagic stroke for females.**

| **Year** | **Cases (no.)** | **Female crude mortality post-haemorrhagic stroke, no. (%)** | | | | |
| --- | --- | --- | --- | --- | --- | --- |
|  |  | **In-hospital** | **30-Day** | **3-Month** | **6-Month** | **1-Year** |
| 2002 | 1,053 | 296 (28.1) | 365 (34.7) | 393 (37.3) | 426 (40.5) | 463 (44.0) |
| 2003 | 1,131 | 334 (29.5) | 414 (36.6) | 448 (39.6) | 472 (41.7) | 494 (43.7) |
| 2004 | 1,053 | 281 (26.7) | 361 (34.3) | 398 (37.8) | 419 (39.8) | 447 (42.5) |
| 2005 | 1,010 | 281 (27.8) | 334 (33.1) | 378 (37.4) | 405 (40.1) | 431 (42.7) |
| 2006 | 1,109 | 323 (29.1) | 400 (36.1) | 426 (38.4) | 447 (40.3) | 476 (42.9) |
| 2007 | 1,038 | 273 (26.3) | 357 (34.4) | 392 (37.8) | 415 (40.0) | 440 (42.4) |
| 2008 | 1,109 | 267 (24.1) | 339 (30.6) | 365 (32.9) | 385 (34.7) | 405 (36.5) |
| 2009 | 1,067 | 262 (24.6) | 347 (32.5) | 381 (35.7) | 400 (37.5) | 426 (39.9) |
| 2010 | 1,069 | 252 (23.6) | 357 (33.4) | 398 (37.2) | 419 (39.2) | 445 (41.6) |
| 2011 | 1,106 | 294 (26.6) | 386 (34.9) | 424 (38.3) | 447 (40.4) | 475 (42.9) |
| 2012 | 1,109 | 269 (24.3) | 356 (32.1) | 387 (34.9) | 412 (37.2) | 445 (40.1) |
| 2013 | 1,058 | 259 (24.5) | 349 (33.0) | 369 (34.9) | 397 (37.5) | 421 (39.8) |
| 2014 | 1,067 | 258 (24.2) | 323 (30.3) | 368 (34.5) | 383 (35.9) | 417 (39.1) |
| 2015 | 1,070 | 214 (20.0) | 314 (29.3) | 340 (31.8) | 354 (33.1) | 382 (35.7) |
| 2016 | 1,166 | 237 (20.3) | 357 (30.6) | 392 (33.6) | 418 (35.8) | 449 (38.5) |
| 2017 | 1,108 | 239 (21.6) | 334 (30.1) | 362 (32.7) | 388 (35.0) | 415 (37.5) |
| **Total** | **17,323** | **43,39 (25.0)** | **5,693 (32.9)** | **6,221 (35.9)** | **6,587 (38.0)** | **7,031 (40.6)** |

**Supplementary Table 6. Cumulative mortality post-haemorrhagic stroke for males.**

| **Year** | **Cases (no.)** | **Male crude mortality post-haemorrhagic stroke, no. (%)** | | | | |
| --- | --- | --- | --- | --- | --- | --- |
|  |  | **In-hospital** | **30-Day** | **3-Month** | **6-Month** | **1-Year** |
| 2002 | 1,169 | 295 (25.2) | 381 (32.6) | 428 (36.6) | 451 (38.6) | 485 (41.5) |
| 2003 | 1,058 | 216 (20.4) | 290 (27.4) | 329 (31.1) | 355 (33.6) | 393 (37.1) |
| 2004 | 1,070 | 240 (22.4) | 302 (28.2) | 344 (32.1) | 367 (34.3) | 407 (38.0) |
| 2005 | 1,049 | 227 (21.6) | 289 (27.6) | 319 (30.4) | 337 (32.1) | 369 (35.2) |
| 2006 | 1,165 | 242 (20.8) | 303 (26.0) | 348 (29.9) | 383 (32.9) | 422 (36.2) |
| 2007 | 1,111 | 237 (21.3) | 297 (26.7) | 342 (30.8) | 365 (32.9) | 403 (36.3) |
| 2008 | 1,125 | 241 (21.4) | 304 (27.0) | 349 (31.0) | 378 (33.6) | 414 (36.8) |
| 2009 | 1,130 | 243 (21.5) | 318 (28.1) | 367 (32.5) | 392 (34.7) | 423 (37.4) |
| 2010 | 1,146 | 216 (18.8) | 291 (25.4) | 331 (28.9) | 364 (31.8) | 396 (34.6) |
| 2011 | 1,096 | 226 (20.6) | 288 (26.3) | 325 (29.7) | 357 (32.6) | 394 (35.9) |
| 2012 | 1,126 | 238 (21.1) | 302 (26.8) | 348 (30.9) | 374 (33.2) | 403 (35.8) |
| 2013 | 1,211 | 240 (19.8) | 329 (27.2) | 386 (31.9) | 406 (33.5) | 448 (37.0) |
| 2014 | 1,096 | 204 (18.6) | 290 (26.5) | 329 (30.0) | 350 (31.9) | 384 (35.0) |
| 2015 | 1,167 | 182 (15.6) | 277 (23.7) | 319 (27.3) | 348 (29.8) | 385 (33.0) |
| 2016 | 1,244 | 192 (15.4) | 290 (23.3) | 330 (26.5) | 357 (28.7) | 406 (32.6) |
| 2017 | 1,147 | 180 (15.7) | 270 (23.5) | 313 (27.3) | 339 (29.6) | 369 (32.2) |
| **Total** | **18,110** | **3,619 (20.0)** | **4,821 (26.6)** | **5,507 (30.4)** | **5,923 (32.7)** | **6,501 (35.9)** |

**Supplementary Table 7. Univariable predictors for in-hospital all-cause mortality.**

| Univariable analysis | Parameters | OR (95% CI) | p-value |
| --- | --- | --- | --- |
| In-hospital death | Age – per-1yr | 1.04 (1.04 – 1.04) | <0.001 |
|  | Male | 0.75 (0.71 – 0.79) | <0.001 |
|  | Surgical evacuation of ICH | 0.37 (0.33 – 0.41) | <0.001 |
|  | Referral source |  | <0.001 |
|  | Emergency Department | 1.00 (reference) |  |
|  | Physician-referred | 0.28 (0.24 – 0.33) | <0.001 |
|  | External hospital-referred | 0.74 (0.67 – 0.81) | <0.001 |
|  | Others | 1.70 (1.29 – 2.23) | <0.001 |
|  | Unknown | 0.62 (0.31 – 1.22) | 0.17 |
|  | Prior stroke | 1.50 (1.38 – 1.63) | <0.001 |
|  | Ischemic heart disease | 2.12 (1.90 – 2.36) | <0.001 |
|  | Prior PCI / CABG | 1.38 (1.16 – 1.62) | <0.001 |
|  | Congestive cardiac failure | 1.97 (1.72 – 2.27) | <0.001 |
|  | Peripheral vascular disease | 1.32 (1.13 – 1.54) | <0.001 |
|  | Prior prosthetic heart valve | 1.25 (0.98 – 1.59) | 0.07 |
|  | Valvular heart disease | 0.80 (0.59 – 1.10) | 0.17 |
|  | Atrial fibrillation/flutter | 1.49 (1.37 – 1.61) | <0.001 |
|  | Hypertension | 1.24 (1.18 – 1.30) | <0.001 |
|  | Hyperlipidaemia | 0.74 (0.65 – 0.84) | <0.001 |
|  | Diabetes | 1.10 (1.03 – 1.19) | 0.008 |
|  | Current smoker | 0.50 (0.46 – 0.56) | <0.001 |
|  | Malignancy | 2.07 (1.84 – 2.33) | <0.001 |
|  | Chronic pulmonary disease | 1.41 (1.21 – 1.65) | <0.001 |
|  | Neurodegenerative disease * | 1.41 (1.28 – 1.55) | <0.001 |
|  | Chronic kidney disease | 1.34 (1.14 – 1.58) | <0.001 |
|  | CCI score – per 1-score † | 1.08 (1.07 – 1.10) | <0.001 |
|  | Year of admission |  | <0.001 |
|  | 2002 | 1.00 (reference) |  |
|  | 2003 | 0.93 (0.81 – 1.06) | 0.26 |
|  | 2004 | 0.90 (0.78 – 1.03) | 0.12 |
|  | 2005 | 0.90 (0.79 – 1.04) | 0.15 |
|  | 2006 | 0.91 (0.80 – 1.04) | 0.18 |
|  | 2007 | 0.86 (0.75 – 0.99) | 0.03 |
|  | 2008 | 0.81 (0.71 – 0.93) | 0.003 |
|  | 2009 | 0.82 (0.72 – 0.94) | 0.005 |
|  | 2010 | 0.74 (0.64 – 0.85) | <0.001 |
|  | 2011 | 0.85 (0.75 – 0.98) | 0.02 |
|  | 2012 | 0.81 (0.71 – 0.93) | 0.002 |
|  | 2013 | 0.78 (0.68 – 0.89) | <0.001 |
|  | 2014 | 0.75 (0.65 – 0.86) | <0.001 |
|  | 2015 | 0.59 (0.51 – 0.69) | <0.001 |
|  | 2016 | 0.60 (0.52 – 0.69) | <0.001 |
|  | 2017 | 0.63 (0.54 – 0.73) | <0.001 |
| CABG, coronary artery bypass graft; CCI, Charlson comorbidity index; CI, confidence interval; ICH, intracranial haemorrhage; OR, odds ratio; PCI, percutaneous coronary interventions.   - Neurodegenerative disease includes dementia, central nervous systemic atrophies, Parkinson’s disease, basal ganglia degeneration, and/or nervous systemic degenerative diseases. - Conditions included in the Charlson Comorbidity Index include myocardial infarction, congestive cardiac failure, peripheral vascular disease, stroke, dementia, chronic pulmonary disease, connective tissue disease, peptic ulcer disease, liver disease (mild vs. moderate to severe), diabetes (with or without organ damage), hemiplegia, moderate to severe renal disease, any tumor (within last 5 years), lymphoma, leukemia, metastatic solid tumor and acquired immunodeficiency syndrome. | | | |

**Supplementary Table 8. Univariable predictors for 1-year all-cause mortality.**

| Univariable analysis | Parameters | HR (95% CI) | p-value |
| --- | --- | --- | --- |
| 1-year death | Age – per-1yr | 1.036 (1.035 – 1.037) | <0.001 |
|  | Male | 0.84 (0.82 – 0.87) | <0.001 |
|  | Surgical evacuation of ICH | 0.42 (0.39 – 0.45) | <0.001 |
|  | Referral source |  | <0.001 |
|  | Emergency Department | 1.00 (reference) |  |
|  | Physician-referred | 0.40 (0.36 – 0.45) | 0.36 |
|  | External hospital-referred | 0.81 (0.76 – 0.86) | 0.76 |
|  | Others | 1.42 (1.19 – 1.70) | 1.19 |
|  | Unknown | 0.51 (0.30 – 0.87) | 0.30 |
|  | Prior stroke | 1.19 (1.13 – 1.26) | <0.001 |
|  | Ischemic heart disease | 1.63 (1.52 – 1.74) | <0.001 |
|  | Prior PCI / CABG | 1.20 (1.07 – 1.34) | 0.002 |
|  | Congestive cardiac failure | 1.75 (1.60 – 1.91) | <0.001 |
|  | Peripheral vascular disease | 1.19 (1.07 – 1.32) | 0.001 |
|  | Prior prosthetic heart valve | 1.12 (0.95 – 1.32) | 0.17 |
|  | Valvular heart disease | 0.86 (0.70 – 1.05) | 0.14 |
|  | Atrial fibrillation/flutter | 1.37 (1.30 – 1.44) | <0.001 |
|  | Hypertension | 1.08 (1.05 – 1.12) | <0.001 |
|  | Hyperlipidaemia | 0.71 (0.65 – 0.78) | <0.001 |
|  | Diabetes | 1.11 (1.06 – 1.16) | <0.001 |
|  | Current smoker | 0.57 (0.53 – 0.61) | <0.001 |
|  | Malignancy | 2.70 (2.53 – 2.88) | <0.001 |
|  | Chronic pulmonary disease | 1.35 (1.23 – 1.49) | <0.001 |
|  | Neurodegenerative disease * | 1.65 (1.56 – 1.75) | <0.001 |
|  | Chronic kidney disease | 1.46 (1.32 – 1.61) | <0.001 |
|  | CCI score – per 1-score † | 1.12 (1.11 – 1.13) | <0.001 |
|  | Year of admission |  | <0.001 |
|  | 2002 | 1.00 (reference) |  |
|  | 2003 | 0.93 (0.85 – 1.02) | 0.14 |
|  | 2004 | 0.92 (0.84 – 1.01) | 0.08 |
|  | 2005 | 0.88 (0.80 – 0.97) | 0.01 |
|  | 2006 | 0.90 (0.82 – 0.98) | 0.02 |
|  | 2007 | 0.89 (0.81 – 0.97) | 0.01 |
|  | 2008 | 0.82 (0.75 – 0.90) | <0.001 |
|  | 2009 | 0.88 (0.80 – 0.96) | 0.005 |
|  | 2010 | 0.85 (0.78 – 0.94) | <0.001 |
|  | 2011 | 0.90 (0.82 – 0.99) | 0.02 |
|  | 2012 | 0.86 (0.78 – 0.94) | 0.001 |
|  | 2013 | 0.87 (0.79 – 0.95) | 0.003 |
|  | 2014 | 0.83 (0.75 – 0.91) | <0.001 |
|  | 2015 | 0.75 (0.68 – 0.83) | <0.001 |
|  | 2016 | 0.78 (0.71 – 0.86) | <0.001 |
|  | 2017 | 0.77 (0.70 – 0.84) | <0.001 |
| CABG, coronary artery bypass graft; CCI, Charlson comorbidity index; CI, confidence interval; HR, hazards ratio; ICH, intracranial haemorrhage; PCI, percutaneous coronary interventions.   - Neurodegenerative disease includes dementia, central nervous systemic atrophies, Parkinson’s disease, basal ganglia degeneration, and/or nervous systemic degenerative diseases. - Conditions included in the Charlson Comorbidity Index include myocardial infarction, congestive cardiac failure, peripheral vascular disease, stroke, dementia, chronic pulmonary disease, connective tissue disease, peptic ulcer disease, liver disease (mild vs. moderate to severe), diabetes (with or without organ damage), hemiplegia, moderate to severe renal disease, any tumor (within last 5 years), lymphoma, leukemia, metastatic solid tumor and acquired immunodeficiency syndrome. | | | |

**Supplementary Table 9. Multivariable predictors for in-hospital all-cause mortality.**

| Parameters | Univariable analysis * | | Multivariable analysis † | |
| --- | --- | --- | --- | --- |
|  | **OR (95% CI)** | **P value** | **OR (95% CI)** | **p-value** |
| Age – per-1yr | 1.04 (1.04 – 1.04) | <0.001 | 1.04 (1.03 – 1.04) | <0.001 |
| Male | 0.75 (0.71 – 0.79) | <0.001 | 0.83 (0.79 – 0.88) | <0.001 |
| Surgical evacuation of ICH | 0.37 (0.33 – 0.41) | <0.001 | 0.46 (0.41 – 0.52) | <0.001 |
| Referral source |  | <0.001 |  | <0.001 |
| Emergency Department | 1.00 (reference) |  | 1.00 (reference) |  |
| Physician-referred | 0.28 (0.24 – 0.33) | <0.001 | 0.35 (0.29 – 0.42) | <0.001 |
| External hospital-referred | 0.74 (0.67 – 0.81) | <0.001 | 0.83 (0.75 – 0.91) | <0.001 |
| Others | 1.70 (1.29 – 2.23) | <0.001 | 1.51 (1.13 – 2.01) | 0.005 |
| Unknown | 0.62 (0.31 – 1.22) | 0.17 | 1.00 (0.50 – 2.02) | 1.00 |
| Prior PCI / CABG | 1.38 (1.16 – 1.62) | <0.001 | 1.18 (0.99 – 1.40) | 0.07 |
| Atrial fibrillation/flutter | 1.49 (1.37 – 1.61) | <0.001 | 1.08 (0.99 – 1.18) | 0.08 |
| Hypertension | 1.24 (1.18 – 1.30) | <0.001 | 0.99 (0.94 – 1.04) | 0.66 |
| Hyperlipidaemia | 0.74 (0.65 – 0.84) | <0.001 | 0.56 (0.49 – 0.64) | <0.001 |
| Current smoker | 0.50 (0.46 – 0.56) | <0.001 | 0.82 (0.74 – 0.92) | <0.001 |
| CCI score – per 1-score † | 1.08 (1.07 – 1.10) | <0.001 | 1.06 (1.05 – 1.08) | <0.001 |
| Year of admission |  | <0.001 |  | <0.001 |
| 2002 | 1.00 (reference) |  | 1.00 (reference) |  |
| 2003 | 0.93 (0.81 – 1.06) | 0.26 | 0.88 (0.77 – 1.01) | 0.08 |
| 2004 | 0.90 (0.78 – 1.03) | 0.12 | 0.87 (0.75 – 1.00) | 0.05 |
| 2005 | 0.90 (0.79 – 1.04) | 0.15 | 0.85 (0.73 – 0.98) | 0.02 |
| 2006 | 0.91 (0.80 – 1.04) | 0.18 | 0.86 (0.74 – 0.99) | 0.03 |
| 2007 | 0.86 (0.75 – 0.99) | 0.03 | 0.81 (0.70 – 0.94) | 0.004 |
| 2008 | 0.81 (0.71 – 0.93) | 0.003 | 0.75 (0.65 – 0.87) | <0.001 |
| 2009 | 0.82 (0.72 – 0.94) | 0.005 | 0.76 (0.66 – 0.87) | <0.001 |
| 2010 | 0.74 (0.64 – 0.85) | <0.001 | 0.66 (0.57 – 0.76) | <0.001 |
| 2011 | 0.85 (0.75 – 0.98) | 0.02 | 0.76 (0.66 – 0.87) | <0.001 |
| 2012 | 0.81 (0.71 – 0.93) | 0.002 | 0.73 (0.63 – 0.84) | <0.001 |
| 2013 | 0.78 (0.68 – 0.89) | <0.001 | 0.68 (0.59 – 0.78) | <0.001 |
| 2014 | 0.75 (0.65 – 0.86) | <0.001 | 0.64 (0.56 – 0.75) | <0.001 |
| 2015 | 0.59 (0.51 – 0.69) | <0.001 | 0.52 (0.45 – 0.60) | <0.001 |
| 2016 | 0.60 (0.52 – 0.69) | <0.001 | 0.51 (0.44 – 0.59) | <0.001 |
| 2017 | 0.63 (0.54 – 0.73) | <0.001 | 0.55 (0.47 – 0.64) | <0.001 |
| CCI, Charlson comorbidity index; CI, confidence interval; OR, odds ratio.   - See Supplementary Table 7 for full univariable analysis results (only univariables with P<0.05 shown). - Only univariables with P<0.05 were included in the multivariable logistic regression analysis. - Conditions included in the Charlson Comorbidity Index include myocardial infarction, congestive cardiac failure, peripheral vascular disease, stroke, dementia, chronic pulmonary disease, connective tissue disease, peptic ulcer disease, liver disease (mild vs. moderate to severe), diabetes (with or without organ damage), hemiplegia, moderate to severe renal disease, any tumor (within last 5 years), lymphoma, leukemia, metastatic solid tumor and acquired immunodeficiency syndrome. | | | | |

**Supplementary Table 10. Multivariable predictors for 1-year all-cause mortality.**

| Parameters | Univariable analysis * | | Multivariable analysis † | |
| --- | --- | --- | --- | --- |
|  | **HR (95% CI)** | **P value** | **HR (95% CI)** | **p-value** |
| Age – per-1yr | 1.036 (1.035 – 1.037) | <0.001 | 1.03 (1.03 – 1.04) | <0.001 |
| Male | 0.84 (0.82 – 0.87) | <0.001 | 0.93 (0.89 – 0.96) | <0.001 |
| Surgical evacuation of ICH | 0.42 (0.39 – 0.45) | <0.001 | 0.49 (0.45 – 0.53) | <0.001 |
| Referral source |  | <0.001 |  | <0.001 |
| Emergency Department | 1.00 (reference) |  | 1.00 (reference) |  |
| Physician-referred | 0.40 (0.36 – 0.45) | 0.36 | 0.51 (0.46 – 0.57) | <0.001 |
| External hospital-referred | 0.81 (0.76 – 0.86) | 0.76 | 0.90 (0.85 – 0.96) | 0.001 |
| Others | 1.42 (1.19 – 1.70) | 1.19 | 1.21 (1.02 – 1.45) | 0.03 |
| Unknown | 0.51 (0.30 – 0.87) | 0.30 | 0.87 (0.51 – 1.47) | 0.60 |
| Prior PCI / CABG | 1.20 (1.07 – 1.34) | 0.002 | 1.04 (0.93 – 1.17) | 0.47 |
| Atrial fibrillation/flutter | 1.37 (1.30 – 1.44) | <0.001 | 1.02 (0.96 – 1.07) | 0.58 |
| Hypertension | 1.08 (1.05 – 1.12) | <0.001 | 0.86 (0.83 – 0.89) | <0.001 |
| Hyperlipidaemia | 0.71 (0.65 – 0.78) | <0.001 | 0.60 (0.54 – 0.65) | <0.001 |
| Current smoker | 0.57 (0.53 – 0.61) | <0.001 | 0.89 (0.83 – 0.96) | <0.001 |
| CCI score – per 1-score ‡ | 1.12 (1.11 – 1.13) | <0.001 | 1.10 (1.09 – 1.11) | 0.002 |
| Year of admission |  | <0.001 |  | <0.001 |
| 2002 | 1.00 (reference) |  | 1.00 (reference) |  |
| 2003 | 0.93 (0.85 – 1.02) | 0.14 | 0.91 (0.83 – 0.99) | 0.04 |
| 2004 | 0.92 (0.84 – 1.01) | 0.08 | 0.91 (0.83 – 1.00) | 0.05 |
| 2005 | 0.88 (0.80 – 0.97) | 0.01 | 0.85 (0.77 – 0.93) | 0.001 |
| 2006 | 0.90 (0.82 – 0.98) | 0.02 | 0.87 (0.79 – 0.95) | 0.002 |
| 2007 | 0.89 (0.81 – 0.97) | 0.01 | 0.86 (0.79 – 0.95) | 0.002 |
| 2008 | 0.82 (0.75 – 0.90) | <0.001 | 0.79 (0.72 – 0.86) | <0.001 |
| 2009 | 0.88 (0.80 – 0.96) | 0.005 | 0.84 (0.77 – 0.92) | <0.001 |
| 2010 | 0.85 (0.78 – 0.94) | <0.001 | 0.80 (0.73 – 0.88) | <0.001 |
| 2011 | 0.90 (0.82 – 0.99) | 0.02 | 0.85 (0.77 – 0.93) | <0.001 |
| 2012 | 0.86 (0.78 – 0.94) | 0.001 | 0.81 (0.74 – 0.89) | <0.001 |
| 2013 | 0.87 (0.79 – 0.95) | 0.003 | 0.80 (0.73 – 0.87) | <0.001 |
| 2014 | 0.83 (0.75 – 0.91) | <0.001 | 0.75 (0.68 – 0.82) | <0.001 |
| 2015 | 0.75 (0.68 – 0.83) | <0.001 | 0.69 (0.62 – 0.76) | <0.001 |
| 2016 | 0.78 (0.71 – 0.86) | <0.001 | 0.69 (0.63 – 0.76) | <0.001 |
| 2017 | 0.77 (0.70 – 0.84) | <0.001 | 0.69 (0.63 – 0.76) | <0.001 |
| CCI, Charlson comorbidity index; CI, confidence interval; HR, hazards ratio; ICH, intracranial haemorrhage.   - See Supplementary Table 8 for full univariable analysis results (only univariables with P<0.05 shown). - Only univariables with P<0.05 were included in the multivariable Cox proportional hazard regression analysis. - Conditions included in the Charlson Comorbidity Index include myocardial infarction, congestive cardiac failure, peripheral vascular disease, stroke, dementia, chronic pulmonary disease, connective tissue disease, peptic ulcer disease, liver disease (mild vs. moderate to severe), diabetes (with or without organ damage), hemiplegia, moderate to severe renal disease, any tumor (within last 5 years), lymphoma, leukemia, metastatic solid tumor and acquired immunodeficiency syndrome. | | | | |

**Supplementary Figure 1. Derivation of study cohort.**

**Legend**

The figure illustrates the derivation of the study cohort.

- Data collected from the Admission-Patient-Data-Collection (APDC) registry, which forms part of the Centre-for-Health-Record-Linkage (CHeReL) database. From the APDC registry, we collected data on consecutive admissions with a primary diagnosis of stroke coded as G45, I60-64 under the International Statistical Classification of Diseases, 10^th^ Revision Australian Modification (ICD-10AM) coding system.

**Supplementary Figure 2. Kaplan-Meier survival curve following admission for haemorrhagic stroke.**

| 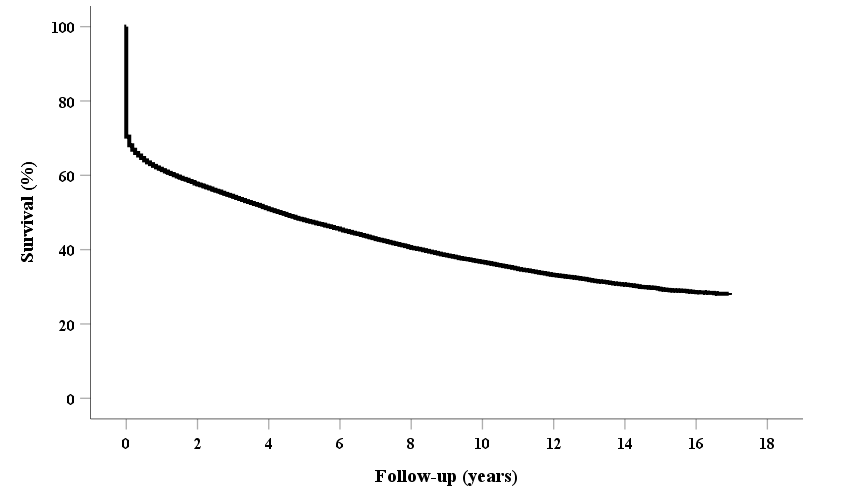   \| **Numbers at risk** \| 35433 \| 19090 \| 14350 \| 10767 \| 7807 \| 5463 \| 3504 \| 1932 \| 580 \| \| --- \| --- \| --- \| --- \| --- \| --- \| --- \| --- \| --- \| --- \| |
| --- | --- | --- | --- | --- | --- | --- | --- | --- | --- | --- |

**Legend**

Figure shows the Kaplan-Meier survival curve of the study cohort following admission for haemorrhagic stroke.
